# YOLO-Tomato: A Robust Algorithm for Tomato Detection Based on YOLOv3

**DOI:** 10.3390/s20072145

**Published:** 2020-04-10

**Authors:** Guoxu Liu, Joseph Christian Nouaze, Philippe Lyonel Touko Mbouembe, Jae Ho Kim

**Affiliations:** 1 Computer Software Institute, Weifang University of Science and Technology, Shouguang 262-700, China; pandalgx@126.com; 2 Department of Electronics Engineering, Pusan National University, Busan 46241, Korea; krxsange@pusan.ac.kr (J.C.N.); lyoneltouko@gmail.com (P.L.T.M.)

**Keywords:** tomato detection, harvesting robots, dense architecture, deep learning

## Abstract

Automatic fruit detection is a very important benefit of harvesting robots. However, complicated environment conditions, such as illumination variation, branch, and leaf occlusion as well as tomato overlap, have made fruit detection very challenging. In this study, an improved tomato detection model called YOLO-Tomato is proposed for dealing with these problems, based on YOLOv3. A dense architecture is incorporated into YOLOv3 to facilitate the reuse of features and help to learn a more compact and accurate model. Moreover, the model replaces the traditional rectangular bounding box (R-Bbox) with a circular bounding box (C-Bbox) for tomato localization. The new bounding boxes can then match the tomatoes more precisely, and thus improve the Intersection-over-Union (IoU) calculation for the Non-Maximum Suppression (NMS). They also reduce prediction coordinates. An ablation study demonstrated the efficacy of these modifications. The YOLO-Tomato was compared to several state-of-the-art detection methods and it had the best detection performance.

## 1. Introduction

Fruits harvesting is very labor intensive and time-consuming work. With the development of artificial intelligence, much of this work can be replaced by a harvesting robot [1]. Harvesting with robots is divided into two steps. First, the fruit detection is performed using a computer vision system. Second, a manipulator is guided to pick the fruits according to the detection results. Of these two steps, fruit detection is the most crucial and challenging. It not only conditions the subsequent operation of the manipulator, but it also determines the detection accuracy. The complicated conditions and nonstructural environment make this task very challenging.

Many researchers have studied fruit detection over the past several decades. Significant improvements have been made [1,2]. Linker et al. [3] used color and texture information to classify green apples. A comparison was performed between a detected circle using this information and a heuristic model to determine the results. An accuracy of 85% was reported. Illumination variation such as direct sunlight and color saturation had a large impact on the results. Wei et al. [4] proposed a color-based segmentation method to extract fruits from background. The OHTA color space was used for segmentation. It may be inferred that the performance is easily affected by the illumination. Kelman et al. [5] proposed a shape analysis method for localization of mature apples. This method first identified the edges in the image using a canny filter. It then detected the edges that correspond to three-dimensional convex objects, using a pre-processing operation and convexity test. They noted that performance is greatly influenced by illumination and leaves that have similar convex surfaces to apples. Payne et al. [6] proposed a color and texture-based algorithm to estimate mango crop yield. The algorithm was a significant improvement over their previous method [7]. However, the situation was constrained by artificial lighting. In addition, the algorithm used a complicated decision process with many fixed thresholds, making it hard to adapt to other fruits or environments. Zhao et al. [8] used a feature images fusion method to recognize mature tomatoes. They adopted the wavelet transformation to fuse the a*-component and I-component from the L*a*b* color space and luminance, in-phase, quadrature-phase (YIQ) color space, respectively. An optimal threshold was then applied on the fusion image to segment tomatoes from the background. They reported a 93% accuracy. Since only color features were used in their study, the results were affected by the illumination.

The growth and development of artificial intelligence techniques have led to more research into applying machine learning to computer vision tasks in agriculture. Lv et al. [9] used a Support Vector Machine (SVM) trained only on RGB color space for fruit and branch identification in natural scenes. They reported that this method obtained an accuracy of 92.4% for fruits, and performed much better than previous threshold-based methods. Nevertheless, the results were prone to be affected by illumination. Kurtulmus et al. [10] conducted experiments using several different classifiers, including statistical classifiers, a neural network, and an SVM for immature peach detection. The circular Gabor filter and principle component analysis were applied for feature extraction. The best accuracy achieved was 84.6%. Performance was restricted to the variations of illumination and occlusion. Yamamoto et al. [11] combined a pixel-based segmentation and a blob-based segmentation strategy for tomato detection. The strategy was based on a decision tree classifier and a random forest classifier. Recall and precision were 80% and 88%. Zhao et al. [12] used a combination of AdaBoost classifier and color analysis for tomato detection. They adopted the Haar-like feature to train the classifier. Although the method can get reasonable results, its speed is relatively low and cannot satisfy the real-time requirement for a harvesting robot. Luo et al. [13] also proposed an AdaBoost and color feature based framework for grape cluster detection. The experiments demonstrated that this method can partly reduce the influence of weather condition, leaves occlusion, and illumination variation. Liu et al. [14] proposed a coarse-to-fine framework for mature tomato detection. Their study adopted an SVM and False Color Removal method. Recall and precision reached 90.00% and 94.41%, respectively. However, the method is not satisfactory for overlapped and occluded tomatoes.

Although traditional machine learning brought great improvements to computer vision, most of the methods are based on handcrafted features which have several drawbacks. First, these features are complicated to design. Second, such features have low-level abstraction and can only adapt to some specific conditions. This results in a weak flexibility. In addition, it is difficult to transfer these methods from one kind of fruit to several others. With the breakthrough of deep learning on computer vision tasks [15,16], these limitations of traditional machine learning were conquered, since features extracted with a deep convolutional neural network (DCNN) are more abstract and are better able to generalize. In particular, the prevalence of big data has paved the way for the applications of deep learning techniques, including agriculture vision tasks [17]. Sa et al. [18] applied the Faster R-CNN [19] detector to fruit detection. The information from the RGB image and Near-Infrared image was used with two fusion methods. This method obtained results better than previous methods. However, it is difficult for the method to detect small fruits, and its speed still needs to be improved for real-time in-field operation of a harvesting robot. Bargoti et al. [20] also proposed a fruit detection model in orchards, based on the Faster R-CNN method. In their report, an F1 score of more than 90% was achieved. Most of the missing fruits came from the case where fruits appear in tight clusters. Rahnemoonfar et al. [21] used a modified Inception-ResNet architecture [22] for fruit counting. This method achieved a 91% average accuracy with real images. However, the method just counted fruit, and did not implement detection. The You Only Look Once (YOLO) models were proposed by Redmon et al. for object detection [23,24,25]. Compared with previous region proposal based detectors [19,26] that perform detection in a two-stage pipeline, the YOLO models directly predict the bounding boxes and their corresponding classes with a single feed forward network. Thus, they can increase the speed significantly while keeping a reasonable accuracy, making them the true sense of real-time detectors. However, there are few studies on fruit detection using the YOLO models.

A detection model based on the DCNN was proposed in this study to detect tomatoes in complex environment conditions. There are two main ideas proposed to improve detection performance.

First, the model incorporated the dense architecture [27] into YOLOv3 [25] to facilitate the reuse of features and make the model learn richer features for tomato representation.

Second, a C-Bbox was proposed to replace the traditional R-Bbox for tomato localization. Since the new bounding boxes match tomatoes better, more accurate IoU could be obtained between a tomato and its corresponding C-Bbox as well as between any two predicted C-Bboxes. They also reduce prediction coordinates.

The experiments demonstrated that the proposed method can achieve a high detection accuracy, and it can also reach a real-time detection speed.

The remainder of this paper is organized as follows. Section 2 describes the theoretical background of detection methods. Section 3 proposes a tomato detection method. Section 4 discusses experimental results, using the proposed method, and Section 5 draws conclusions from this paper.

## 2. Theoretical Background

### 2.1. YOLO Series

The YOLOv3 [25] is one of the state-of-the-art object detection methods that evolved from YOLO [23] and YOLOv2 [24]. Unlike Faster R-CNN [19], it is a single-stage detector that formulates the detection problem as a regression problem.

**Figure 1:**
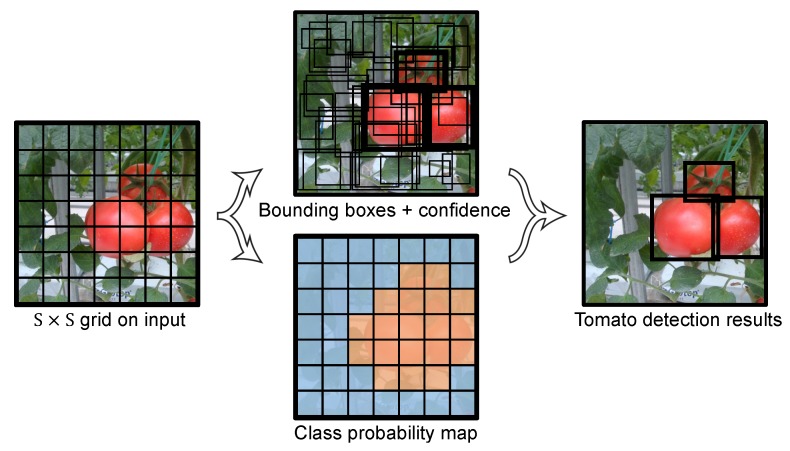
YOLO model detection.

The YOLO framework is illustrated in Figure 1. The main concept is to divide the input image into a S×S grid, and to make detections in each grid cell. Each cell predicts B bounding boxes along with the confidence of these boxes. The confidence can reflect whether an object exists in the grid cell and, if it does, the IoU of the ground truth (GT) and predictions. The confidence can be formulated in Equation (1):

(1) Confidence=Pr(Object)×IoU(GT,pred)

where $Pr\left(Object\right)\in \left[0,1\right]$.

Each grid cell also predicts *C* class probabilities for the object. In total, (5+C) values are predicted by each cell: *x*, *y*, *w*, *h*, confidence and *C* class probabilities. (*x*, *y*) represent the center coordinates of the box, and (*w*, *h*) represent the width and height of the box, respectively.

Inspired by Faster R-CNN, YOLOv2 borrowed an idea of the prior anchors for detection, which can simplify the problem and ease the learning process of the network. It also draws on some other concepts such as batch normalization [28] and skip connection [29]. Compared to YOLO, YOLOv2 significantly improves localization and recall.

Based on YOLOv2, Redmon proposed a more powerful detector—YOLOv3. Motivated by the feature pyramid framework, like in [30], YOLOv3 predicts objects in three different scales. This can remedy the object size variation problem.

### 2.2. Densely Connected Block

**Figure 2:**
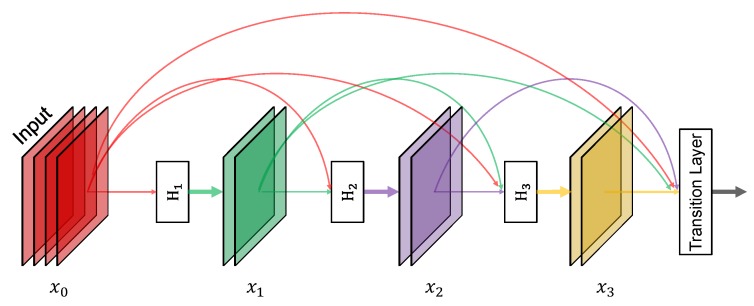
A 4-layer dense block. Each layer takes all preceding feature-maps as input and serves as input for all subsequent layers. $H_{i}$ denotes the operation BN-ReLU-Conv_1×1_-BN-ReLU-Conv_3×3_.

To better facilitate the reuse of features, Huang [27] proposed a densely connected convolutional network (DenseNet). The characteristic of DenseNet is that, for each layer in a dense block, it takes output of all the preceding layers as input and serves as input for all subsequent layers. Thus, for *L* layers, the network has $\frac{L\left(L+1\right)}{2}$ connections. With this property, the DenseNet can significantly relieve the gradient vanishing problem, make better reuse of features, and facilitate feature propagation. Figure 2 shows an example of a 4-layer dense block. As a consequence, the *l*th layer $x_{l}$ takes as input the feature maps of all preceding $\left(l-1\right)$ layers, $x_{1},\ldots {,x}_{l-1}$, as illustrated in Equation (2):

(2) $$x_{l}=H_{l}\left[x_{0},x_{1},\ldots ,x_{l-1}\right])$$

where $\left[x_{0},x_{1},\ldots ,x_{l-1}\right]$ represents the concatenation of the output feature maps generated in layers 0 to l−1, and $H_{l}\left(\cdot \right)$ is a combinatory function of several sequential operations, i.e., BN [28], ReLU [31], and convolution. In this study, $H_{l}\left(\cdot \right)$ denotes BN-ReLU-Conv_1×1_-BN-ReLU-Conv_3×3_.

### 2.3. The Non-Maximum Suppression for Merging Results

Since object detectors usually perform detection in a sliding window form [32] or in many densely distributed prior anchors [33], there may be several detections corresponding to the same object. The NMS method is used to remove the redundant detections and to find the best match.

NMS is widely used in many types of tasks [32,33] and has proved its efficiency. The process is summarized in Algorithm 1. Since R-Bboxes are commonly used to localize objects, the IoU of adjacent R-Bboxes is adopted in NMS for merging the results.
**Algorithm 1.** The pseudo code of NMS method**Input:** $B=\{b_{1},\cdots ,b_{N}\}$, $C=\{c_{1},\cdots ,c_{N}\}$, $\lambda_{nms}$
    B is the list of initial detection boxes
    C contains corresponding detection confidences
    $\lambda_{nms}$ is the NMS threshold**Output:** List of final detection boxes O1: O←{}2: **while**
 B≠∅
**do**3:  m←argmaxC4:  $O\leftarrow O\cup b_{m};\;B\leftarrow B-b_{m};\;C\leftarrow C-c_{m}$5:  **for**
$\;b_{i}\in B$
**do**6:   **if**$\;IoU\left(b_{m},b_{i}\right)\geq \lambda_{nms}\;$
**then**7:    $B\leftarrow B-b_{i};\;C\leftarrow C-c_{i}$8:   **end if**9:  **end for**10: **end while**

## 3. Materials and Methods

### 3.1. Image Acquisition

The tomato datasets used in this paper were collected in a period from December 2017 to November 2019 in Vegetable High-tech Demonstration Park, Shouguang, China. The images were captured using a digital camera (Sony DSC-W170, Tokio, Japan) with a 3648 × 2056-pixel resolution. All the images were taken under natural daylight conditions, including several disturbances: illumination variation, occlusion, and overlap.

**Figure 3:**
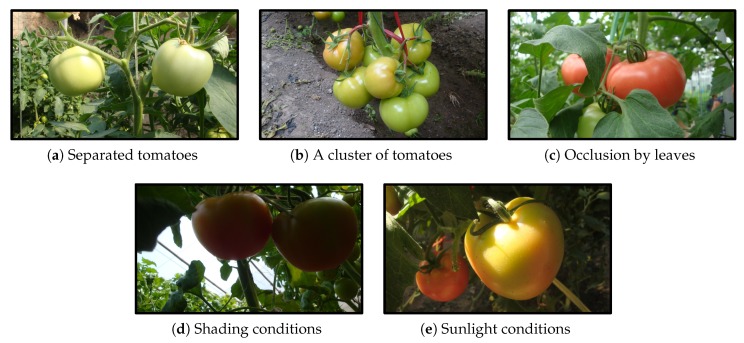
Tomato samples with different growing circumstances: (**a**) two separated tomato, (**b**) a cluster of tomatoes, (**c**) occlusion case, (**d**) shading conditions, and (**e**) sunlight conditions.

A total of 966 tomato images were captured and divided into a training set and a test set. The training set consisted of 725 images which contained 2553 tomatoes, and the remaining 241 images which included 912 tomatoes made up the test set. Figure 3 shows some samples from the dataset under different environments.

### 3.2. Image Augmentation

The data augmentation technique was used in this study. While training, before input into the model, each image was randomly sampled by one of the following options:

– the entire original image
– scaling and cropping

**Figure 4:**
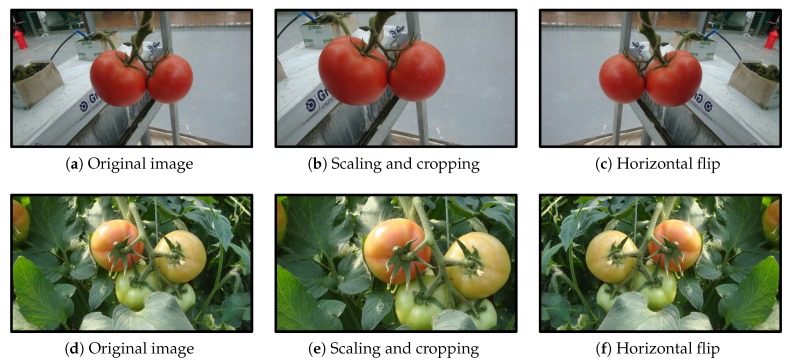
Some examples of image augmentation operations: (**left**): original images, (**middle**): scaling and cropping, and (**right**): horizontal flip.

For the scaling and cropping operation, the image was first scaled with a random factor falling in the range [1.15, 1.25]. Then, a patch with the same size as the original image was randomly cropped from the scaled image. After the sampling step, each image was horizontally flipped with a probability of 0.5. Some examples of the augmentation are shown in Figure 4.

### 3.3. The Proposed YOLO-Tomato Model

**Figure 5:**
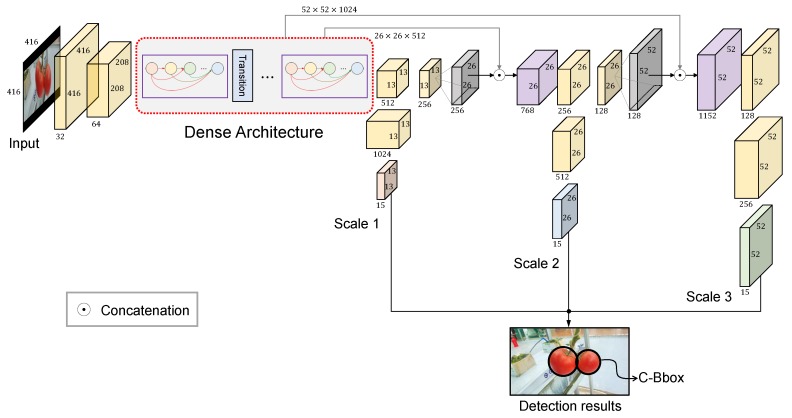
An overview of the proposed model.

**Figure 6:**
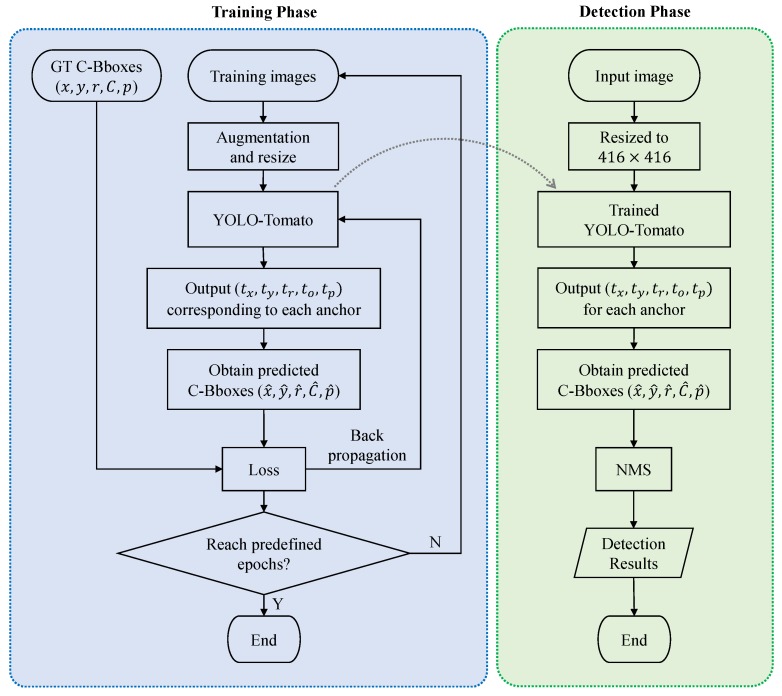
A flowchart of training and detection process of YOLO-Tomato.

An overview of the proposed tomato detection model is shown in Figure 5. On the basis of the YOLOv3 model, a dense architecture was incorporated for better feature reuse and representation. Furthermore, a C-Bbox was proposed instead of the traditional R-Bbox. The C-Bbox can match the shape of a tomato better, consequently making a more precise localization. Moreover, the C-Bbox can derive a more accurate IoU between the predictions, which plays an important role in the NMS process, and thus improve the detection results. The proposed model is called YOLO-Tomato. Figure 6 shows a flowchart of training and detection process of YOLO-Tomato. Section 3.4 and Section 3.5 present more details.

### 3.4. Dense Architecture for Better Feature Reuse

It has been proven in [27] that direct connection between any two layers allows the feature reuse throughout the networks and therefore helps to learn more compact and accurate models. To better reuse the features for tomato detection, a densely connected architecture is incorporated into the YOLOv3 framework, as in Figure 5. With this modification, the extracted features can be utilized more efficiently, especially for those from low-level layers, which can be expected to improve the accuracy of detection.

**Figure 7:**
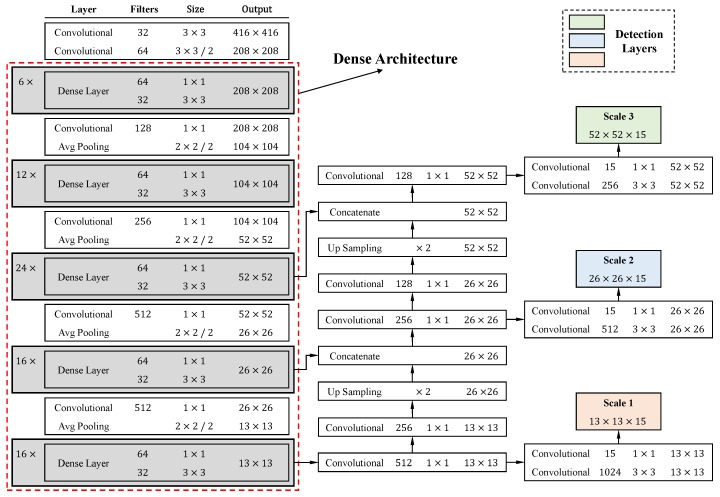
Dense architecture of the proposed model.

A specification of dense architecture used in this study is shown in Figure 7. There are five dense blocks in this architecture, which consists of 6, 12, 24, 16, and 16 dense layers, respectively. For each dense layer, a 1×1 bottleneck layer [29] and a 3×3 convolutional layer are stacked together. To make the model more compact, a transition layer was placed between (any) two consecutive dense layers. The structure of a dense block is illustrated in Figure 2. Owing to the direct connection between any two layers inside the dense block, the network can learn more rich features and improve the representation of tomatoes. In the original YOLOv3 model, there are six convolutional layers in front of each of the detection layers. Due to the better use of features by dense architecture, the original six layers were pruned to two layers before each detection layer, by removing the first four layers. The results of the experiment demonstrate the effectiveness of the proposed architecture in Section 4.

### 3.5. Circular Bounding Box

For general object detection tasks, e.g., Pascal VOC [34] and COCO [35], an R-Bbox is usually adopted to localize the target since the shape of objects varies with the classes. However, when focusing on a specific task, a customized shape of a bounding box could be used to improve the detection performance. In this study, since the detection target is tomato (a circle shape), a C-Bbox is proposed. Due to the better match of tomatoes and the C-Bboxes, it is believed the proposed C-Bbox has two main advantages when compared to the traditional R-Bbox. On one hand, the IoU of two predicted C-Bboxes is more accurate than that of R-Bboxes, which plays an important role in the NMS process. On the other hand, the C-Bbox has less parameters than R-Bbox, making it easier for the CNN model to regress from the prior anchors to the predictions. The C-Bbox is illustrated in more detail in the following.

#### 3.5.1. IoU of Two C-Bboxes

**Figure 8:**
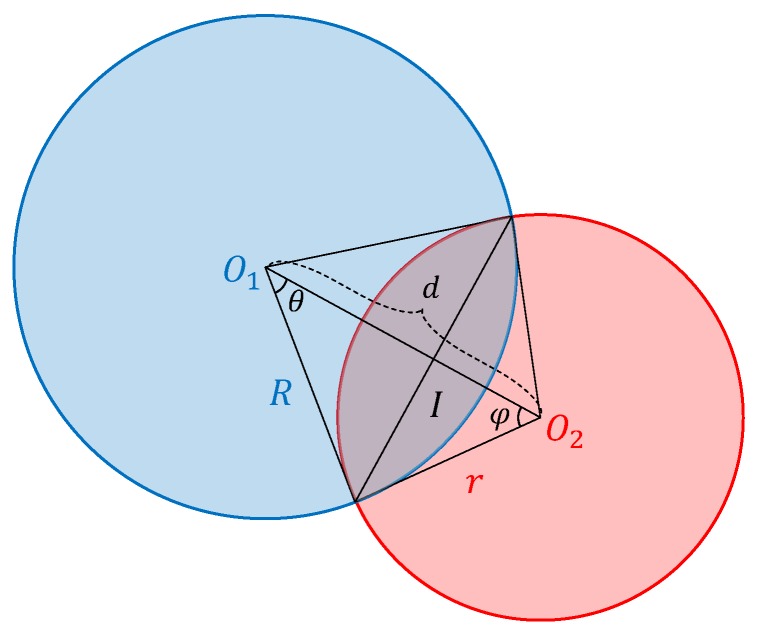
Overlap of two C-Bboxes.

Given two circles $O_{1}$ and $O_{2}$, which are overlapped as in Figure 8, it can be shown that, if their radii *R* and *r* satisfy Equation (3), the overlap area $A_{overlap}$ can be derived as in Equation (4):

(3) |R−r|≤d≤|R+r|

where d is the distance of the centers of the two circles $O_{1}$ and $O_{2}$.

(4) $$A_{overlap}=\theta R^{2}+\varphi r^{2}-\frac{1}{2}R^{2}\sin2\theta -\frac{1}{2}r^{2}\sin2\varphi$$

where θ and φ can be derived as in Equations (5) and (6):

(5) $$\theta =\arccos\frac{R^{2}+d^{2}-r^{2}}{2Rd}$$

(6) $$\varphi =\arccos\frac{r^{2}+d^{2}-R^{2}}{2rd}$$

Then, the IoU of $O_{1}$ and $O_{2}$ can be calculated in Equation (7):

(7) $$IoU\left(O_{1},O_{2}\right)=\frac{A_{overlap}}{\pi R^{2}+\pi r^{2}-A_{overlap}}$$

If the circle $O_{2}$ is entirely contained in circle $O_{1}$, their IoU can be calculated as in Equation (8):

(8) $$IoU\left(O_{1},O_{2}\right)=\frac{r^{2}}{R^{2}}$$

#### 3.5.2. C-Bbox Location Prediction and Loss Function

**Figure 9:**
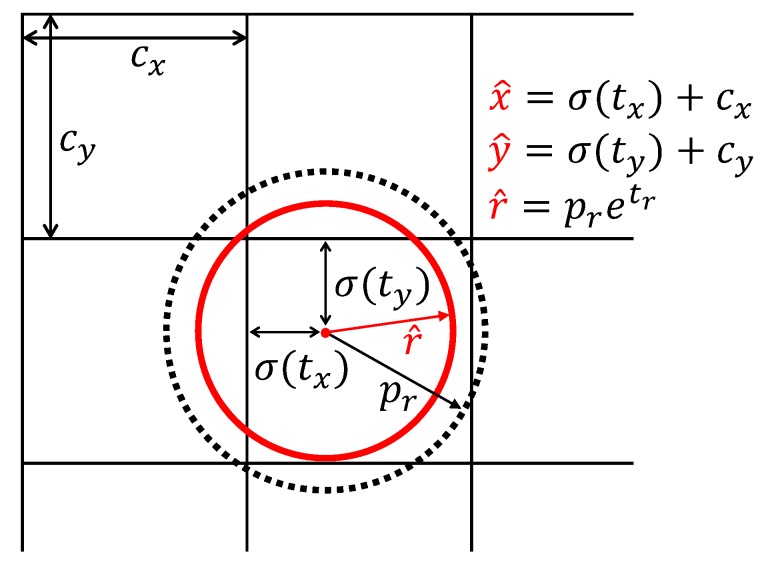
C-Bbox prediction. The black dotted circle indicates the prior anchor, and the red circle is the prediction.

Since the R-Bbox was replaced with the proposed C-Bbox for tomato detection, the prior anchors would also be set as circular anchors. As a result, the modified model predicts only three coordinates for each C-Bbox – $t_{x}$, $t_{y}$, $t_{r}$. If the grid cell has an offset of ($c_{x}$, $c_{y}$) from the top left corner of the image, and the prior anchors have a radius of $p_{r}$, the predictions will be calculated as in Equations (9)–(11). Figure 9 is an illustration of the C-Bbox prediction:

(9) $$\hat{x}=\sigma \left(t_{x}\right)+c_{x}$$

(10) $$\hat{y}=\sigma \left(t_{y}\right)+c_{y}$$

(11) $$\hat{r}=p_{r}e^{t_{r}}$$

where σ(·) is sigmoid function.

Accordingly, the loss function used for C-Bbox prediction was adjusted as in Equation (12):

(12) $$\begin{array}{cc} Loss=\lambda_{\mathrm{coord}} & \sum_{i=1}^{S^{2}}\sum_{j=1}^{B}{𝟙}_{i,j}^{\mathrm{obj}}\left[{\left(x_{i}-{\hat{x}}_{i}\right)}^{2}+{\left(y_{i}-{\hat{y}}_{i}\right)}^{2}\right]\\ \; & \;+\lambda_{\mathrm{coord}}\sum_{i=1}^{S^{2}}\sum_{j=1}^{B}{𝟙}_{i,j}^{\mathrm{obj}}{\left(\sqrt{r_{i}}-\sqrt{{\hat{r}}_{i}}\right)}^{2}\\ \; & +\sum_{i=1}^{S^{2}}\sum_{j=1}^{B}{𝟙}_{i,j}^{\mathrm{obj}}\left(-C_{i}\log{\hat{C}}_{i}\right)\\ \; & +\lambda_{\mathrm{noobj}}\sum_{i=1}^{S^{2}}\sum_{j=1}^{B}{𝟙}_{i,j}^{\mathrm{noobj}}\left[-\left(1-C_{i}\right)\log\left(1-{\hat{C}}_{i}\right)\right]\\ \; & +\sum_{i=1}^{S^{2}}\sum_{j=1}^{B}{𝟙}_{i,j}^{\mathrm{obj}}\sum_{c\in \mathrm{classes}}\left[-p_{i}\left(c\right)\log{\hat{p}}_{i}\left(c\right)-\left(1-p_{i}\left(c\right)\right)\log\left(1-{\hat{p}}_{i}\left(c\right)\right)\right] \end{array}$$

where $\hat{x}$, $\hat{y}$, $\hat{r}$ are the center coordinates and radius of the C-Bbox. $\hat{C}$ denotes the confidence for prediction, and $\hat{p}\left(c\right)$ is the predicted class probability. *x*, *y*, *r*, *C*, and p(c) are the counterparts for GT. ${𝟙}_{i,j}^{\mathrm{obj}}$ indicates that the *j*th bounding box in grid cell *i* matches the object in the cell, while ${𝟙}_{i,j}^{\mathrm{noobj}}$ indicates the remaining non-matched bounding boxes. $S^{2}$ denotes the S×S grid cells, and *B* is the number of prior anchors in each cell. To remedy the imbalance problem between positive and negative samples, $\lambda_{coord}$ and $\lambda_{noobj}$ are set to 5 and 0.5, respectively, as in [23].

### 3.6. Experimental Setup

In this study, the experiments were conducted on a computer that has Intel i5 (Santa Clara, CA, USA), 64-bit 3.30 GHz quad-core CPUs, and a NVIDIA GeForce GTX 1070Ti GPU.

The model receives images of 416×416 pixels as inputs. Due to GPU memory constraints, the batch size was set to 8. The model was trained for 160 epochs with an initial learning rate of $10^{-3}$, which was then divided by 10 after 60 and 90 epochs. The momentum and weight decay were set to 0.9 and 0.0005, respectively.

A series of experiments were conducted to evaluate the performance of the proposed method. The indexes for evaluation of the trained model are defined as follows:

(13) $$Recall=\frac{TP}{TP+FN}$$

(14) $$Precision=\frac{TP}{TP+FP}$$

where TP, FN, and FP are abbreviations for true positives (correct detection), false negatives (miss), and false positives (false detection).

To better show the comprehensive performance of the model, F_1_ score was adopted as a trade-off between the recall and precision, defined in Equation (15):

(15) $$F_{1}=\frac{2\times Recall\times Precision}{Recall+Precision}$$

Another evaluation metric for object detection—Average Precision (AP) [34,36]—was also used in this study. It can show the overall performance of a model under different confidence thresholds, and is defined as follows:

(16) $$AP=\sum_{n}\left(r_{n+1}-r_{n}\right)p_{interp}\left(r_{n+1}\right)$$

with

(17) $$p_{interp}\left(r_{n+1}\right)=\max_{\tilde{r}:\tilde{r}\geq r_{n+1}}p\left(\tilde{r}\right)$$

where $p(\tilde{r})$ is the measured precision at recall $\tilde{r}$.

## 4. Results and Discussion

### 4.1. Average IoU Comparison of C-Bbox and R-Bbox

**Figure 10:**
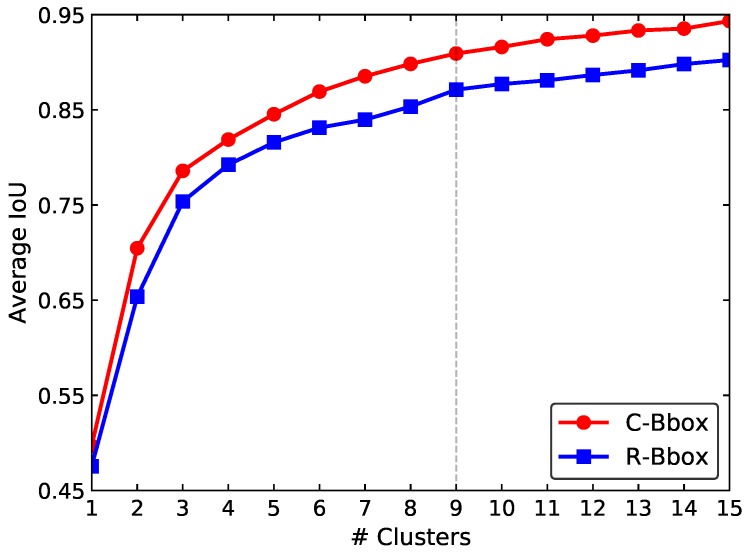
Clustering anchor dimensions of R-Bbox and C-Bbox. The k-means clustering was used to get the prior anchors. As in [24], the IoU was adopted instead of the Euclidean distance as the evaluation metric. As indicated by the dotted vertical line, nine clusters were adopted as the prior anchors, and were then divided into three parts and assigned to each of the three scales for detection.

In this study, to evaluate the performance of the proposed C-Bbox, as in [24], the average IoU between each type of the bounding boxes and GTs of the training set was calculated and compared. As shown in Figure 10, the average IoU of the C-Bbox is higher than that of the R-Bbox for all of the cluster numbers. This is as expected since the C-Bbox intrinsically matches the shape of tomatoes better than the R-Bbox. This advantage of the C-Bbox makes it easier for the detection model to regress from the prior anchors to the GTs. In this study, nine clusters were adopted as prior anchors for tomato detection.

### 4.2. Ablation Study on Different Modifications

**Figure 11:**
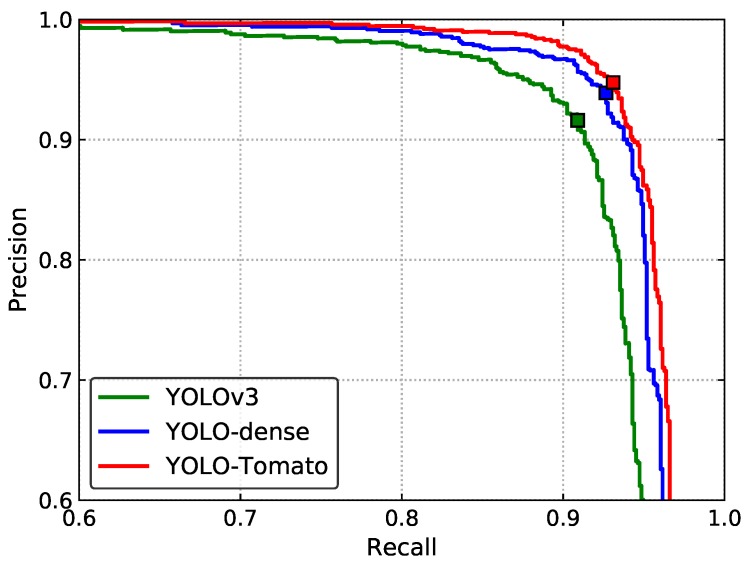
P–R curves of different methods for ablation study. The markers indicate the points where recall and precision are obtained when the prediction confidence threshold equals 0.8.

An ablation analysis of the effect of the dense architecture and C-Bbox was studied. For convenience, incorporation of only the dense architecture is called YOLO-dense. Figure 11 shows the precision–recall curves (P–R curves). The markers indicate the points where recall and precision are obtained when the confidence threshold equals 0.8. The corresponding values are shown in Table 1. The table shows that incorporation of dense architecture brought a significant rise of both the recall and precision, consequently resulting in an improvement of the F_1_ score from 91.24% to 93.26%. This demonstrates the effectiveness of the feature reuse of the dense connection, which presents a richer representation of tomatoes. Furthermore, if the R-Bbox was replaced by the proposed C-Bbox, the F_1_ score of the model would increase about 0.65%, mainly benefiting from the better match between the C-Bbox and tomatoes. In accordance with the P–R curves in Figure 11, the AP was improved with each modification, as shown in Table 1.

### 4.3. The Network Visualization

**Figure 12:**
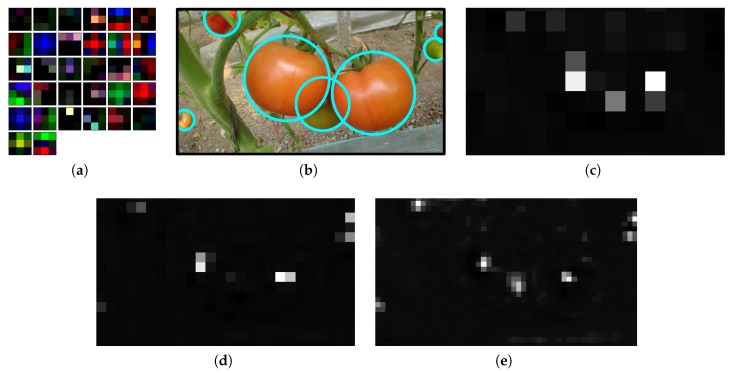
(**a**) the 32 3×3 filters of Conv1 of the network, (**b**) the input image (cyan circles are marked manually for a better visualization), and (**c**–**e**), one of the feature activations from the 80th, 86th, and 92th convolutional layers, respectively.

Although it is difficult to understand the mechanism of the deep neural network clearly, some visual clues are shown in this section that DCNN could capture some discriminative features. Figure 12a shows 32 filters of the first convolutional layer of the model. One can see that some filters learned edge information of different directions, while other filters presented some color features, such as red, green, and brown, etc. To show the effectiveness of the model for tomato detection, some of the feature maps obtained from different convolutional layers (80, 86, and 92) are shown in Figure 12c–e. These layers correspond to different detection scales. The corresponding input image is shown in Figure 12b with manual marking (cyan circles) of the tomatoes for a better visualization. The first feature map shows that only the regions corresponding to the headmost tomatoes are activated. Although occluded by other two tomatoes, the region of the middle tomato was still activated weakly. The top and right regions where smaller tomatoes are present are activated in the second feature map. The region for the smallest tomato in the bottom left is activated in the third feature map. Combining the results from different scales, all of the tomatoes are detected by the model.

### 4.4. Impact of Training Dataset Size on Tomato Detection

**Figure 13:**
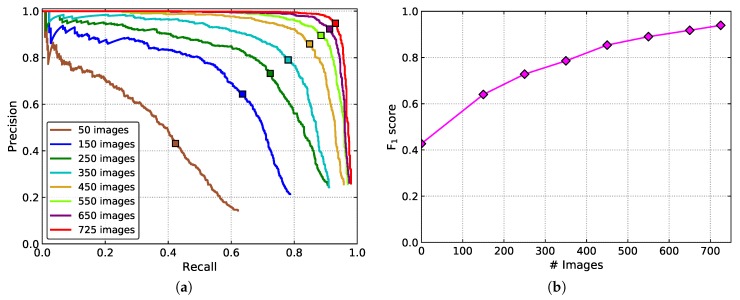
(**a**) P–R curves, and (**b**) the F_1_ scores of the models trained with different size of datasets.

The influence of the size of the dataset for tomato detection was also analyzed. Eight different sizes of training datasets were set up for evaluation. In addition to the whole training set, 50, 150, 250, 350, 450, 550, and 650 tomato images were randomly selected from the training set to form the new datasets. Recall, precision, F_1_ score, and the AP are shown in Table 2. The corresponding P–R curves are shown in Figure 13.

From the results, one can conclude that the performance of the detection model improves with the increase of the dataset size. As shown in Figure 13b, if the number of images is less than 450, the F_1_ score increases rapidly with the growth of the number. When the size of the dataset exceeds 450, the boost speed of the performance slows down gradually and tends to saturate.

### 4.5. Performance of the Proposed Model under Different Lighting Conditions

**Figure 14:**
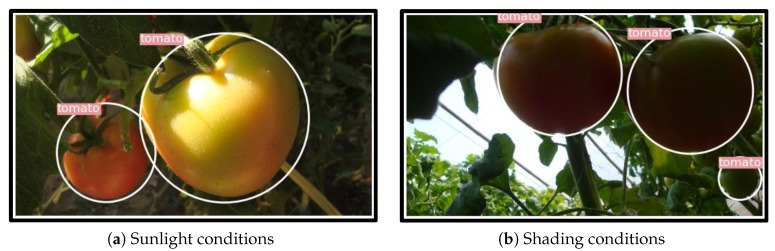
Some examples of the detection results under different lighting conditions.

Robustness of the proposed model to different illumination conditions was examined in this study. Among all the tomatoes, 487 were presented under sunlight. The remaining 425 were in shading conditions. In Table 3, the correct identification rate (or recall) for the sunlight conditions reaches 93.22%, which is comparable to that of shading conditions (92.94%). Among all the detected tomatoes under sunlight conditions, 5.22% of them were falsely detected and belong to the background, while for the shading conditions, this rate is 5.28%. The results indicate that the proposed model is robust to illumination variation, which is a key factor for harvesting robot to operate under complex environments. Some examples of detection results are shown in Figure 14.

### 4.6. Performance of the Proposed Model under Different Occlusion Conditions

To evaluate the performance of the proposed model under different occlusion conditions, the tomatoes were divided into slight and severe occlusion cases according to their occlusion level. Severe cases included tomatoes being blocked by leaves, stems, or other tomatoes by more than 50% degree. Others were identified as slight cases.

**Figure 15:**
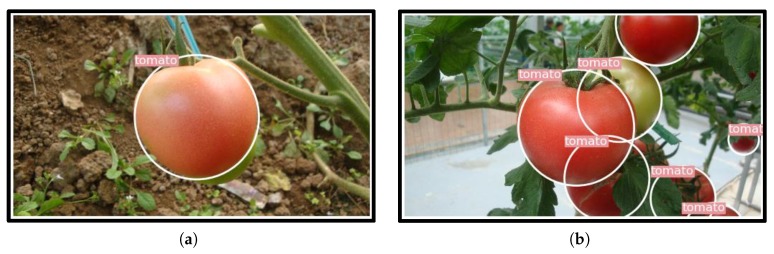
Some missed detection results due to severe occlusion by leaves or other tomatoes: (**a**) the green tomato which was largely covered by the red one was not detected, and (**b**) the red tomato was missed due to severe occlusion by leaves, stems and other tomatoes.

The results are shown in Table 4. Under slight occlusion conditions, 94.58% of the tomatoes were detected. That was about 4.5% higher than the severely occluded ones. Under severe occlusion cases, the presence of the tomatoes was quite different from that of the intact ones, accounting for the loss of some semantic information. Some examples are shown in Figure 15. Of note, due to the severe overlap or occlusion, the green tomato in the left image and the red tomato in the right image were both missed by the proposed detector. Nevertheless, this is not a vital issue since, for harvesting robots, the detection and picking processes were operated alternately. Hidden tomatoes would appear after picking the front tomatoes. The detection accuracy is expected to improve with the incorporation of contextual information like calyx. Another potential improvement would occur when zooming in on candidate regions by approaching the cameras to the tomatoes, and then only performing detection on these regions.

### 4.7. Comparison of Different Algorithms

To validate the performance of the proposed YOLO-Tomato model, other state-of-the-art detection methods were evaluated for comparison—YOLOv2 [24], YOLOv3 [25], and Faster R-CNN [19].

**Figure 16:**
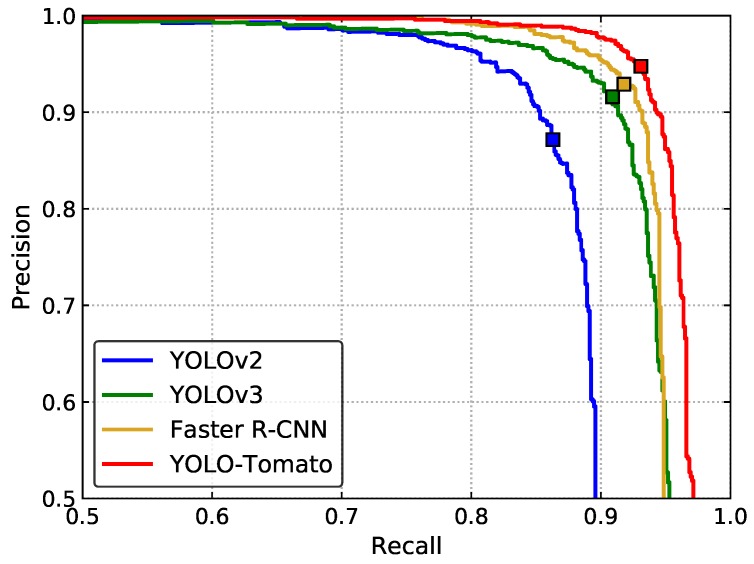
P–R curve for different detection methods.

Figure 16 shows the P–R curves of several methods on the test set. The recall, precision, F_1_ score, and AP of different methods are shown in Table 5. The proposed YOLO-Tomato shows the best detection performance among all the methods. This method achieved the highest recall, precision, and F_1_ score. Its AP reached 96.40%, which is higher than that of YOLOv2, YOLOv3, and Faster R-CNN, indicating the superiority of the proposed method. The detection time of YOLO-Tomato is 0.054 s per image on average. It is about 0.17 s less than Faster R-CNN. This indicates that the model could perform tomato detection in real time, which is important for harvesting robots.

Furthermore, the Wilcoxon signed-ranks test [37] was performed to compare different methods with an objective to see whether the proposed method outperformed other methods with a statistical significance.

**Figure 17:**
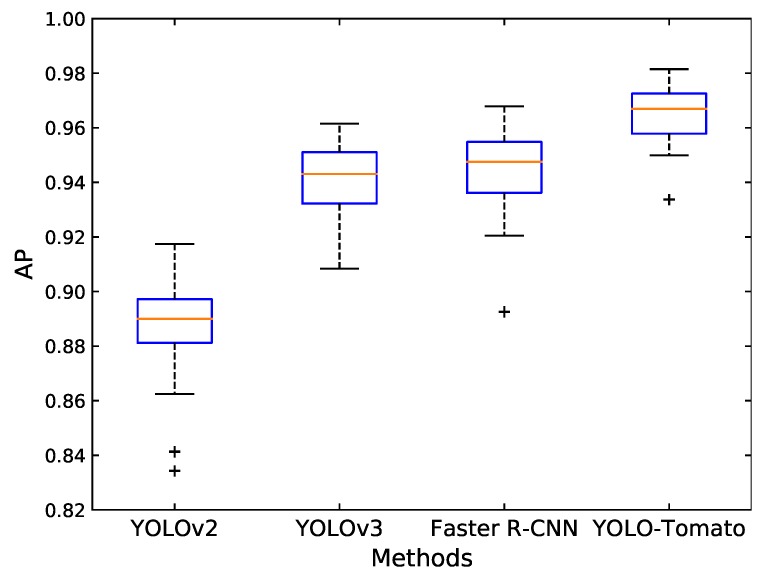
The AP of different methods performed on the 30 sub-datasets displayed using boxplots. The red lines indicate the median values of AP, and ”+” indicates the outliers.

Thirty sub-datasets were randomly sampled from the original test set, each with 80 images. Each model was applied on the 30 sub-datasets, and the corresponding AP was calculated. The *p*-values for each pair of methods were obtained using the Wilcoxon signed-ranks test. Table 6 shows the results. The results are analyzed at a significance level of 0.05, i.e., the null hypothesis—“there is no significant difference between the two methods” is rejected if *p*-value ≤ 0.05. From Table 6, one can conclude that all pairs of methods have significant differences. Figure 17 shows the boxplot diagrams for the AP of different methods performed on the 30 sub-datasets. It is observed that the proposed YOLO-Tomato performed better than other methods.

## 5. Conclusions and Future Work

This study proposed the use of a YOLO-Tomato detector for tomato detection, based on the YOLOv3 model. This method is able to reduce some of the influence of illumination variation, overlap, and occlusion. This was achieved through two approaches. The first incorporated the dense architecture for feature extraction, which can make better reuse of features and help to learn more accurate models. The second replaced the traditional R-Bbox with a proposed C-Bbox, better matching the tomato shape and providing more precise IoU for the NMS process, and reducing prediction coordinates.

Different experiments were conducted to verify the performance of the proposed method. An ablation study of the dense architecture and C-Bbox showed the effectiveness of each modification. Incorporating dense architecture could contribute about 2% improvement on F_1_ score. Based on the dense architecture, a further adoption of C-Bbox could contribute another 0.65% improvement on the F_1_ score. Experiments under different illumination and occlusion conditions were also conducted. The proposed model showed comparable results under both sunlight and shading conditions. This indicates the robustness of the model to illumination variation.

The model shows a divergence under different occlusion conditions. Under slight occlusion conditions, the correct identification rate of YOLO-Tomato reaches 94.58%. This is more than 4% higher than that of severe conditions. This was mainly attributed to the loss of semantic information by severe occlusion.

The proposed method performed better than three other state-of-the-art methods. The superiority of this method demonstrated that it can be applied to harvesting robots for tomato detection.

In future work, the contextual information around tomatoes will be utilized to improve the detection performance, especially for severely occluded tomatoes. In addition, information about tomato ripeness will be studied and incorporated to detect tomatoes in different growing stages.

## Figures and Tables

**Table 1:** Ablation study of dense architecture and circular bounding box.

Methods	Dense Architecture	C-Bbox	Recall (%)	Precision (%)	F_1_ (%)	AP (%)
YOLOv3			90.89	91.60	91.24	94.06
YOLO-dense	**✓**		92.65	93.88	93.26	95.56
YOLO-Tomato	**✓**	**✓**	93.09	94.75	93.91	96.40

**Table 2:** The detection performance with different size of datasets.

Dataset Size	Recall (%)	Precision (%)	F_1_ (%)	AP (%)
50	42.32	43.13	42.72	34.37
150	63.59	64.37	63.98	60.58
250	72.37	73.25	72.81	74.61
350	78.07	79.02	78.54	81.51
450	84.87	85.90	85.38	89.94
550	88.48	89.56	89.02	93.57
650	91.23	92.34	91.78	95.07
725	93.09	94.75	93.91	96.40

**Table 3:** The detection results of the proposed method under different lighting conditions.

Illumination	Tomato Count	Correctly Identified		Falsely Identified		Missed	
		Amount	Rate (%)	Amount	Rate (%)	Amount	Rate (%)
Sunlight	487	454	93.22	25	5.22	33	6.78
Shading	425	395	92.94	22	5.28	30	7.06

**Table 4:** The performance of the proposed model under different occlusion conditions.

Occlusion Condition	Tomato Count	Correctly Identified		Falsely Identified		Missed	
		Amount	Rate (%)	Amount	Rate (%)	Amount	Rate (%)
Slight case	609	576	94.58	22	3.68	33	5.42
Severe case	303	273	90.10	25	8.39	30	9.90

**Table 5:** A comparison of different tomato detection methods.

Methods	Recall (%)	Precision (%)	F_1_ (%)	AP (%)	Time (ms)
YOLOv2 [24]	86.18	87.24	86.71	88.46	30
YOLOv3 [25]	90.89	91.60	91.24	94.06	45
Faster R-CNN [19]	91.78	92.89	92.33	94.37	231
YOLO-Tomato	93.09	94.75	93.91	96.40	54

**Table 6:** The *p*-value obtained by the Wilcoxon signed-rank test for each pair of detection methods.

	YOLOv2	YOLOv3	Faster R-CNN	YOLO-Tomao
YOLOv2		0.000	0.000	0.000
YOLOv3	0.000		0.047	0.000
Faster R-CNN	0.000	0.047		0.000
YOLO-Tomato	0.000	0.000	0.000	

## Abbreviations

The following abbreviations are used in this manuscript:

YOLO	You Only Look Once
R-Bbox	Rectangular Bounding Box
C-Bbox	Circular Bounding Box
IoU	Intersection-over-Union
NMS	Non-Maximum Suppression
SVM	Support Vector Machine
DCNN	Deep Convolutional Neural Network
GT	Ground Truth
DenseNet	Dense Convolutional Network
TP	True Positive
FN	False Negative
FP	False Positive
P–R curve	Precision–Recall curve
AP	Average Precision
